# A rare case of delayed duodenal perforation due to an over-the-scope clip

**DOI:** 10.1055/a-2285-3137

**Published:** 2024-04-09

**Authors:** Yujiro Kawakami, Shinji Yoshii, Masahiro Taniguchi, Yoshiharu Masaki, Taro Sugawara, Yasutoshi Kimura, Hiroshi Nakase

**Affiliations:** 1 92187 Department of Gastroenterology and Hepatology, Sapporo Medical University School of Medicine, Sapporo, Japan; 2 92187 Department of Surgical Pathology, Sapporo Medical University School of Medicine, Sapporo, Japan; 3 92187 Department of Surgery, Surgical Oncology and Science, Sapporo Medical University School of Medicine, Sapporo, Japan

A 53-year-old man was referred to our department because of jaundice. We performed endoscopic retrograde cholangiopancreatography, which revealed an ampullary carcinoma. Endoscopic biliary and pancreatic stenting were performed ( Fig. 1 ); however, 3 days later, the patient developed a fever and abdominal pain. Computed tomography (CT) revealed a duodenal perforation due to stent deviation ( Fig. 2 ). Esophagogastroduodenoscopy confirmed the duodenal perforation opposite the papilla that had been caused by a pancreatic stent. We deployed an over-the-scope (OTS) clip (Ovesco Endoscopy AG, Tübingen, Germany) for closure of the perforated site ( Fig. 3 ; Video 1 ). Subsequently, we performed percutaneous drainage for a retroperitoneal abscess that formed, extending from the site of the perforation to the pelvis. The retroperitoneal abscess did not improve, even after 3 weeks of drainage. Contrast imaging of the percutaneous drainage tube revealed leakage from the side of the OTS clip deployment site ( Fig. 4 ). We diagnosed the patient as having a delayed perforation due to the OTS clip and performed pancreaticoduodenectomy. Histopathological findings showed perforation of all layers in the area of the OTS clip ( Fig. 5 ).

**Fig. 1:**
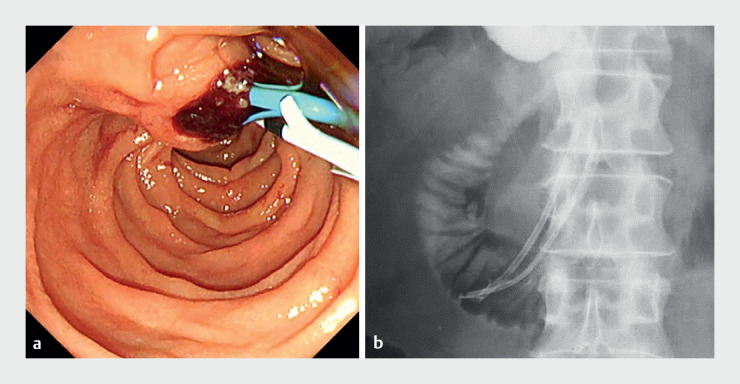
Images taken after endoscopic biliary and pancreatic stenting had been performed.

**Fig. 2:**
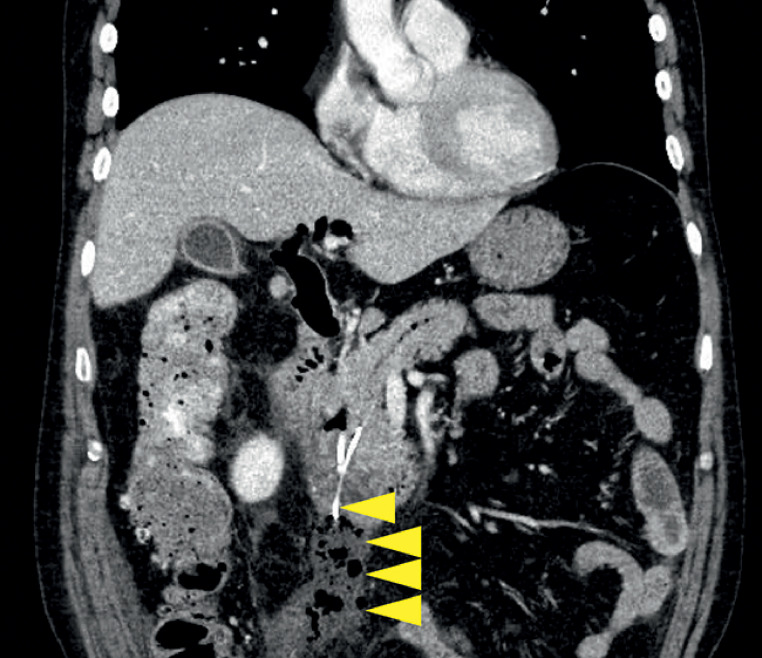
Computed tomography image showing evidence of duodenal perforation due to stent deviation (arrow heads).

**Fig. 3:**
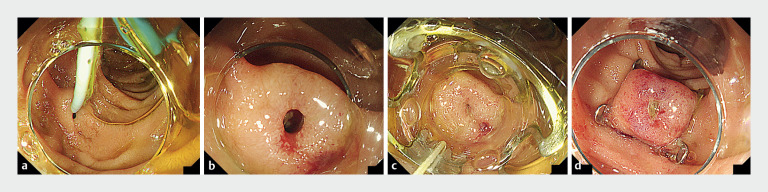
Endoscopic images showing: **a** , **b** a perforation of the duodenal wall opposite the papilla that had been caused by a pancreatic stent; **c** , **d** deployment of an over-the-scope clip (Ovesco Endoscopy AG, Tübingen, Germany) to achieve closure of the perforation.

A delayed perforation is identified in the duodenum after placement of an over-the-scope clip to treat a perforation that had been caused by a pancreatic stent.Video 1

**Fig. 4:**
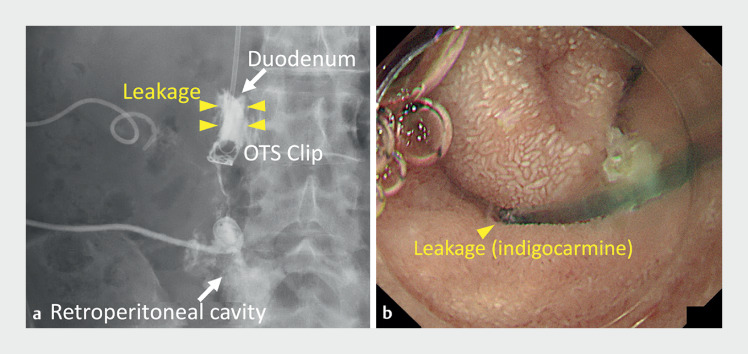
Images following injection of contrast into the percutaneous drainage tube showing: **a** on fluoroscopic imaging, leakage from the side of the deployment site of the over-the-scope (OTS) clip (arrow heads); **b** on endoscopic view, leakage of indigo carmine from the percutaneous drainage tube near the OTS clip.

**Fig. 5:**
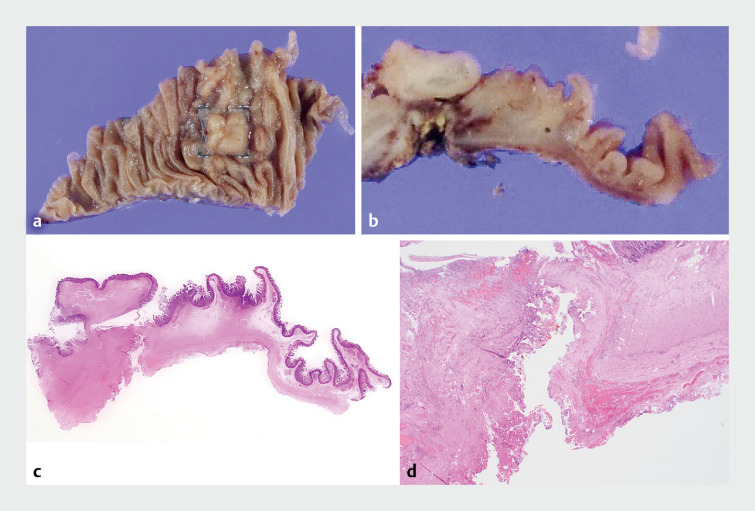
Pathological findings showing perforation of all layers of the duodenal wall in the area of the over-the-scope clip on: **a** , **b** the formalin-fixed specimen; **c** , **d** the hematoxylin and eosin (H&E)-stained section.

This is the first case report of delayed perforation caused by an OTS clip. The OTS clip is effective and safe for complicated bleeding and the closure of perforations of the gastrointestinal tract 1 . With its large jaw width and greater strength, it provides a much tighter closure than conventional endoclips 2 ; however, previous studies have demonstrated that tight closure can cause mucosal ischemia 3 . In the present case, delayed perforation could have been caused by additional conditions: (i) tissue fragility owing to malnutrition, and (ii) exposure to pancreatic juice and bile in the duodenum. In such conditions, endoscopists should keep in mind the possibility of delayed perforation due to an OTS clip.

Endoscopy_UCTN_Code_CPL_1AH_2AL

## Notes

Endoscopy E-Videos https://eref.thieme.de/e-videos
*E-Videos* is an open access online section of the journal *Endoscopy* , reporting on interesting cases and new techniques in gastroenterological endoscopy. All papers include a high-quality video and are published with a Creative Commons CC-BY license. Endoscopy E-Videos qualify for HINARI discounts and waivers and eligibility is automatically checked during the submission process. We grant 100% waivers to articles whose corresponding authors are based in Group A countries and 50% waivers to those who are based in Group B countries as classified by Research4Life (see: https://www.research4life.org/access/eligibility/ ). This section has its own submission website at https://mc.manuscriptcentral.com/e-videos .
